# Anaemia as a Determinant of Cognitive Dysfunction in Peritoneal Dialysis Patients: Evidence from a Single-Centre Study

**DOI:** 10.3390/medicina62010195

**Published:** 2026-01-16

**Authors:** Mira Novković Joldić, Branimirka Aranđelović, Jelena Vojnović, Dario Novaković, Blanka Slavik, Milica Knežević, Dragana Milutinović

**Affiliations:** 1 Faculty of Medicine, University of Novi Sad, 21000 Novi Sad, Serbia; branimirka.arandjelovic@mf.uns.ac.rs (B.A.); jelena.vojnovic@mf.uns.ac.rs (J.V.); 910040d23@mf.uns.ac.rs (D.N.); 401371@mf.uns.ac.rs (B.S.); 910080d24@mf.uns.ac.rs (M.K.); 2 Clinic for Nephrology and Clinical Immunology, University Clinical Centre of Vojvodina, 21000 Novi Sad, Serbia; 3 Clinic for Gastroenterology and Hepatology, University Clinical Centre of Vojvodina, 21000 Novi Sad, Serbia

**Keywords:** cognitive dysfunction, peritoneal dialysis, anaemia, risk factors

## Abstract

*Background and Objectives:* Cognitive disorders are a significant health problem in patients undergoing peritoneal dialysis and can profoundly impair both quality of life and treatment outcomes. Early identification of risk factors for the development of cognitive disorders in this population is therefore essential. This study aimed to (1) determine the prevalence of cognitive dysfunction in patients on peritoneal dialysis, (2) examine its association with sociodemographic characteristics, and (3) assess whether anaemia is associated with cognitive dysfunction in these patients. *Materials and Methods:* A cross-sectional study was conducted in November 2024 at the University Clinical Centre of Vojvodina, Clinic for Nephrology and Clinical Immunology, and included 36 patients on peritoneal dialysis. The Montreal Cognitive Assessment (MoCA) was used to evaluate cognitive function, while a structured questionnaire was used to collect sociodemographic data. Anaemia was determined based on haemoglobin levels. *Results:* Cognitive dysfunction was present in 69.4% of patients on peritoneal dialysis, while anaemia, as indicated by haemoglobin values, was present in 58.3% of the sample. Older age, rural residence, and lower haemoglobin levels were significantly associated with cognitive dysfunction in patients on peritoneal dialysis. *Conclusions:* Preserved cognitive function is a key prerequisite for the adequate implementation of peritoneal dialysis and for maintaining patients’ quality of life. The findings indicate the need for further research to identify effective strategies for preventing and treating anaemia, a factor associated with cognitive dysfunction in this patient population.

## 1. Introduction

Chronic kidney disease (CKD) is a major public health problem affecting more than 10% of the world’s population [1]. It is defined as a clinical syndrome characterised by a gradual, irreversible loss of nephrons, leading to impairment of all kidney functions, excretory, endocrine, and metabolic [2]. In the terminal stage of chronic renal failure, it is necessary to initiate kidney replacement therapies such as hemodialysis (HD), peritoneal dialysis (PD), or kidney transplantation [3].

Hemodialysis replaces renal function using an artificial semipermeable membrane, enabling the exchange of substances between the dialysis fluid and the patient’s blood [4]. Peritoneal dialysis is a kidney replacement therapy modality in which solutes and fluids are transported across the peritoneal membrane by diffusion and osmosis. The two main PD modalities are continuous ambulatory peritoneal dialysis (CAPD) and automated peritoneal dialysis (APD). Compared with HD, PD offers several advantages: it is more convenient, can be performed at home by the patient, enables greater flexibility for travel and fluid intake, and allows for longer preservation of residual renal function [5].

Anaemia is a frequent complication in patients with chronic renal failure. It results from shortened erythrocyte lifespan, chronic blood loss, and inadequate erythropoiesis, most commonly due to insufficient erythropoietin production [2]. Reduced erythropoietin levels and anaemia can lead to brain hypoxia and a decline in cognitive function [3]. Anaemia negatively affects quality of life in patients with chronic renal failure through symptoms such as fatigue, dyspnoea, reduced physical capacity, and impaired cognitive performance. It can influence cognitive function directly, by reducing cerebral perfusion and oxygenation, and indirectly by increasing the risk of cerebrovascular disease. Anaemia is also associated with a higher prevalence of cardiovascular diseases in this population [2]. Correction of anaemia in patients with end-stage chronic renal failure improves quality of life by reducing dyspnoea, improving physical activity, and enhancing cognitive functioning [1].

Cognitive dysfunction (CD) is a clinical syndrome characterised by a decline in at least two domains of mental function, including episodic memory, language, attention, orientation, visuospatial and executive functions, and abstract thinking [6]. CD may range from mild deficits that do not interfere with activities of daily living (ADL) to dementia, defined as persistent and progressive cognitive impairment that compromises ADL and patient independence [3]. The reported prevalence of CD in patients with CKD ranges from 10% to 40%, depending on the definition of cognitive impairment, the assessment tools used, and the stage of CKD [1].

Multiple risk factors are shared between CKD and cognitive disorders, including arterial hypertension, diabetes mellitus, older age, and hyperlipidaemia. In addition, CKD-related factors such as anaemia, hypoxaemia, accumulation of uraemic toxins, oxidative stress, and chronic inflammation may further contribute to cognitive decline [3]. For this reason, patients with advanced CKD should undergo regular cognitive assessment by an appropriate specialist, and if needed, receive timely therapeutic interventions. Cognitive disorders can adversely affect disease prognosis and treatment success by impairing patients’ ability to understand and process information, participate in shared decision-making, adhere to complex treatment regimens, and follow recommended dietary restrictions [7].

Cognitive functioning is a key determinant of a patient’s capacity to perform daily activities. Consequently, CD leads to a decline in quality of life, which is a key outcome measure in the treatment of patients with CKD, where the goals of care are less focused on cure and more on adaptation to physical limitations, lifestyle changes, and long-term medical treatment. Improved quality of life and self-efficacy are associated with better outcomes in dialysis patients, including favourable laboratory parameters, improved cognitive and emotional functioning, lower mortality and hospitalisation rates, and better adherence [8].

A meta-analysis comparing the effects of HD and PD on cognitive function suggests that PD may be a more suitable treatment modality in terms of cognitive outcomes, as patients on HD have been shown to exhibit worse cognitive performance and a higher risk of dementia compared with patients on PD [3]. Furthermore, because PD can be performed at home, patients treated with PD generally report a higher quality of life and self-efficacy than those treated with HD [8]. However, PD, as a home-based modality, requires that patients correctly and consistently perform all procedural steps, placing considerable demands on cognitive abilities [4]. Patients on PD who have cognitive dysfunction may be unable to safely continue treatment with this modality, as errors during dialysis can lead to serious, potentially life-threatening complications [9].

Early identification of risk factors for cognitive disorders in patients with end-stage chronic renal failure is therefore crucial for supporting self-care, ensuring safe PD implementation, and improving quality of life. Since patients’ cooperation and adherence to treatment depend substantially on their cognitive abilities, nurses should be aware of the possibility of CD when planning and providing care and should adapt their educational strategies and support accordingly [2].

In this context, the present study aimed to:

(a) Determine the frequency of cognitive dysfunction in patients on peritoneal dialysis;
(b) Examine the association between cognitive dysfunction and patients’ sociodemographic characteristics;
(c) Assess whether anaemia is associated with cognitive dysfunction in these patients.

## 2. Materials and Methods

### 2.1. Study Design and Participants

This single-centre cross-sectional study was conducted in November 2024 at the Clinic for Nephrology and Clinical Immunology at the University Clinical Centre of Vojvodina. The study population consisted of adult patients undergoing peritoneal dialysis. Inclusion criteria were patients on peritoneal dialysis for more than 3 months, aged 18 to 80 years, and able to provide informed consent. Exclusion criteria were treatment with hemodialysis and a history of kidney transplantation. A total of 36 patients on peritoneal dialysis met the inclusion criteria and agreed to participate in the study.

### 2.2. Research Instruments

In line with the study aims, a structured general questionnaire was developed to collect sociodemographic data, including sex, year of birth, place of residence, level of education, marital status, and underlying renal disease.

Cognitive function of the patients was assessed using the Serbian version of the Montreal Cognitive Assessment (MoCA), which was administered during a regular check-up at the Clinic in the morning hours. The MoCA is a brief, 30-point screening tool for mild cognitive impairment that covers eight cognitive domains: executive functions, attention and concentration, memory, language, visuospatial skills, conceptual thinking, calculation, and orientation. The test typically takes about 10 min to complete. In accordance with standard scoring recommendations, one point was added to the total score for participants with fewer than 12 years of formal education. Cognitive dysfunction was defined as a MoCA score ≤ 25.

Data on clinical, biochemical, and peritoneal dialysis parameters were obtained from patients’ medical records. Anaemia was determined based on haemoglobin (Hb) concentration. A complete blood count was performed using a SYMEX XYN-1000 analyser (Kobe, Japan), which utilises flow cytometry. Physiological haemoglobin values were considered to be 138–172 g/L in men and 120–160 g/L in women, and values < 110 g/L were classified as anaemia.

### 2.3. Data Processing

Data was analysed using descriptive and inferential statistics, in accordance with the research objectives. Continuous variables are presented as means and standard deviations or as medians and interquartile ranges (Q1–Q3), depending on the distribution of the data. Non-parametric tests were used to analyze the association between the MoCA score and sociodemographic variables. The normality of the MoCA score distribution was checked with the Shapiro–Wilk test.

Spearman’s correlation coefficient (ρ) was used to examine the association between age and MoCA score. Differences in MoCA score between binary categorical variables (gender, residence, and marital status) were analyzed using the Mann–Whitney U test (U). Differences in MoCA scores by education level were examined using the Kruskal–Wallis test (H).

Pearson’s and point-biserial correlation analyses were conducted to examine associations between cognitive performance (MoCA scores), haemoglobin levels, and erythropoietin therapy (coded as a dichotomous variable). Pearson correlation analysis was applied only when the assumption of normality was met. Statistical analyses were performed using IBM SPSS Statistics, version 25. A two-tailed *p*-value < 0.05 was considered statistically significant.

### 2.4. Ethical Considerations

The study was approved by the Ethics Committee of the University Clinical Centre of Vojvodina (approval No. 00-383) and by the clinic’s management. All procedures were conducted in accordance with the Declaration of Helsinki. Before inclusion, all patients were informed about the study’s purpose and procedures and provided written informed consent to participate.

## 3. Results

### 3.1. General Characteristics of the Patient

Of the 36 patients included in the study, the majority were male (n = 22, 61.1%). The most represented age category was ≥60 years (n = 17, 47.2%). Most patients lived in an urban area (n = 21, 58.3%). Regarding educational level, 29 patients (80.5%) had completed secondary education, 5 (13.9%) had higher education, and 2 (5.6%) had primary education or lower.

Most patients were married (n = 23, 63.9%), while 13 (36.1%) were divorced. Arterial hypertension was the most prevalent comorbidity (n = 27, 75.0%), followed by diabetes mellitus (n = 6, 16.7%) and polycystic kidney disease (n = 3, 8.3%). Sociodemographic and clinical characteristics of the patients are presented in Table 1.

### 3.2. Prevalence of Cognitive Dysfunction in Patients on Peritoneal Dialysis

According to the MoCA cut-off value (≤25 points), cognitive dysfunction was present in 25 patients (69.4%), while 11 patients (30.6%) had scores > 25, indicating no cognitive dysfunction by this criterion.

Among patients with cognitive dysfunction, the most frequent MoCA score was 22 points (n = 7, 19.4%), followed by 23 points (n = 6, 16.7%). In the group without cognitive dysfunction, the most common scores were 26 and 30 points, each observed in 4 patients (11.1%).

Overall, MoCA scores in the sample ranged from 17 to 30 points, with a mean of 23.97, indicating reduced cognitive performance in a substantial proportion of patients (Table 2).

### 3.3. Association Between Cognitive Dysfunction and Sociodemographic Characteristics

The association between sociodemographic characteristics and cognitive status (MoCA score) was examined using non-parametric statistical tests (Table 3).

A statistically significant negative correlation was found between age and MoCA score (ρ = −0.48, *p* = 0.003), indicating that older patients have lower MoCA scores. A significant difference was also observed for place of residence (U = 223.0, *p* = 0.035), indicating that patients from rural areas have lower MoCA scores than those from urban areas. Gender, education, and marital status did not show statistically significant differences in patients on peritoneal dialysis (Table 3).

### 3.4. Prevalence of Anaemia in Patients on Peritoneal Dialysis

Analysis of haemoglobin parameters showed that 21 patients (58.3%) met the criteria for anaemia, while 15 patients (41.7%) had haemoglobin values within the reference range. Regarding erythropoietin therapy, 12 patients (33.3%) were not receiving erythropoietin, whereas 24 patients (66.7%) were on erythropoietin treatment (Table 4).

### 3.5. Association of Anaemia with Cognitive Dysfunction in Patients on Peritoneal Dialysis

The relationship between haemoglobin levels, erythropoietin therapy, and cognitive performance (MoCA score) was examined using Pearson’s and point-biserial correlation analyses (Table 5). A significant positive correlation was found between haemoglobin level and MoCA score (r = 0.487, *p* = 0.020), indicating that higher haemoglobin levels were associated with better cognitive performance. In addition, a significant positive correlation was observed between haemoglobin level and erythropoietin therapy (r_pβ_ = 0.332, *p* = 0.048), reflecting the expected association between treatment and haemoglobin status. No statistically significant correlation was found between erythropoietin therapy and MoCA score (r_pβ_ = −0.124, *p* = 0.472). These findings suggest that anaemia, as reflected by lower haemoglobin levels, is associated with poorer cognitive performance in patients on peritoneal dialysis (Table 5).

## 4. Discussion

In this study, we assessed the prevalence of cognitive dysfunction in patients on peritoneal dialysis using the MoCA test and examined its association with sociodemographic characteristics and anaemia as potential factors influencing cognitive function.

According to the MoCA results, cognitive dysfunction was present in 25 of 36 patients (69.4%) on peritoneal dialysis. Thus, nearly two-thirds of our sample showed impaired cognitive performance, whereas only about one-third had preserved cognition according to the applied cut-off. These findings are consistent with previous reports. A one-year cross-sectional study conducted in China among 98 patients reported a prevalence of cognitive dysfunction of 69.39% in PD patients, also assessed using the MoCA [10]. Lower prevalences have been reported in studies that used other instruments. In a one-year cross-sectional study of 445 patients, Yu Li et al. used the 3MS test and found cognitive impairment in 23.6% of PD patients [11].

In contrast, a cross-sectional study from Poland, including 18 patients and using the ACE-III, reported a prevalence of 33% [6]. Differences in prevalence estimates across studies are likely related to heterogeneity in study design, sample size, population characteristics, and, particularly, the cognitive screening tools used and their respective cut-off values. The MoCA is known to be more sensitive for detecting mild cognitive impairment than some other instruments [2], which may contribute to the higher prevalence observed in our similar studies.

Regarding sociodemographic factors, our findings indicate that age is significantly associated with cognitive status. As age increases, MoCA scores decrease modestly but significantly, as expected and consistent with previous research showing age-related decline in specific cognitive domains among PD and CKD patients [12,13]. Age is a well-established non-modifiable risk factor for cognitive impairment, and our results reinforce the need for particular attention to older patients on peritoneal dialysis.

In our study, place of residence also emerged as a sociodemographic characteristic significantly associated with MoCA scores. Patients living in rural areas had lower MoCA scores compared to those from urban areas. This difference may be a consequence of lower availability of health services, lower level of social and cognitive stimulation, different living conditions or socioeconomic differences that characterize rural areas. This finding highlights the need for further research to explore how specific contextual factors related to urban and rural living conditions (e.g., social support, environmental exposures, lifestyle) influence cognitive functions in PD patients.

Contrary to some previous studies [12,13,14], we did not find a statistically significant association between educational level and cognitive dysfunction in our sample of PD patients. While higher education is generally considered a protective factor due to its contribution to cognitive reserve, our result suggests that in this small and clinically specific population, other socio-economic or clinical factors may play a more important role in maintaining cognitive function than formal education alone. Given that cognitive impairment is common among PD patients, further studies on larger samples are needed to investigate relationships between cognitive status and broader socio-economic determinants not covered in this study, such as social support, health behaviours, income level and employment status.

In our sample, the prevalence of anaemia in PD patients, defined by haemoglobin levels, was 58.3%. This indicates a relatively high anaemia prevalence, despite 66.7% of patients being treated with erythropoietin at the time of laboratory assessment. Comparisons with other studies show considerable variability. A cross-sectional study from Brazil, including 120 PD patients, reported an anaemia prevalence of 28%, with 87.5% of patients receiving erythropoietin therapy [15]. Conversely, a retrospective three-month study from Spain found that 89% of 343 PD patients had anaemia, while 72.9% were on erythropoietin treatment [16]. These differences likely reflect heterogeneity in study designs, definitions of anaemia, target haemoglobin levels, dialysis adequacy, iron status and local treatment protocols. Nevertheless, all cited studies, including ours, confirm that anaemia is a very common complication in patients on peritoneal dialysis.

Regarding the impact of anaemia on cognitive function, our results indicate that haemoglobin levels are significantly associated with cognitive performance in PD patients. We found a positive correlation between haemoglobin level and MoCA score (r = 0.487, *p* = 0.02), suggesting that higher haemoglobin levels are associated with better cognitive functioning. Adequate haemoglobin levels contribute to optimal oxygen delivery to brain tissue, which is crucial for normal neuronal activity and cognitive processes. In contrast, chronic hypoxia due to low haemoglobin levels may impair cerebral oxygenation and negatively affect neurological function, contributing to cognitive decline [3]. This aligns with other studies. For example, a prospective study from Turkey involving 112 patients identified low haemoglobin levels as a risk factor for cognitive decline [17]. Similarly, the two-year prospective study by Shea YF et al. on 206 PD patients reported anaemia among the potential risk factors for the development of cognitive impairment [18].

Our findings therefore support the hypothesis that anaemia is an important, potentially modifiable factor associated with cognitive dysfunction in patients on peritoneal dialysis. Given the high prevalence of cognitive impairment in this population, routine cognitive screening and regular hemoglobin monitoring with the use of adequate therapy could contribute to reducing the risk of cognitive decline and improving clinical outcomes and quality of life of these patients. Further research is needed to identify effective strategies for the prevention and treatment of cognitive dysfunction in PD patients, including optimising anaemia management and implementing comprehensive interventions that address both clinical and psychosocial determinants of cognitive health.

### Limitations

Although our study is among the few that specifically examine the impact of anaemia on cognitive function in peritoneal dialysis patients and thus contributes to the understanding of mental functioning in this population, several limitations must be acknowledged.

First, the study was conducted at a single nephrology clinic in the Republic of Serbia, with a relatively small sample of 36 patients. This limits the generalisability of the findings to the broader PD patient population and reduces statistical power to detect more subtle associations. Second, the cross-sectional design does not allow for conclusions about causality or the temporal sequence of events; we cannot determine whether anaemia precedes cognitive decline or vice versa, nor how changes in haemoglobin over time affect cognitive trajectories.

Third, cognitive function was assessed using a single screening instrument (MoCA). Although MoCA is a sensitive and widely used tool for detecting mild cognitive impairment, it does not provide a full neuropsychological profile. Additionally, we did not analyse the potential influence of some important confounding factors, such as dialysis adequacy, inflammatory markers, iron status, depression, sleep disorders or detailed socio-economic indicators, which may also be related to cognitive function, while allowing for the application of a multivariate model.

Longitudinal, multicentre studies with larger samples, comprehensive cognitive assessments and a broader set of clinical and socio-economic variables are needed to confirm and extend our findings.

## 5. Conclusions

In this single-centre cross-sectional study conducted in November 2024 at the University Clinical Centre of Vojvodina, Clinic for Nephrology and Clinical Immunology, we assessed the prevalence of cognitive dysfunction in patients on peritoneal dialysis and examined its association with sociodemographic characteristics and anaemia. The main findings can be summarised as follows:

- Cognitive dysfunction is highly prevalent among patients on peritoneal dialysis, affecting 69.4% of the studied sample according to the MoCA cut-off (≤25 points).
- There is a significant negative association between cognitive performance and age, indicating that older patients tend to have lower MoCA scores. Place of residence was also significantly associated with cognitive status, with patients living in rural areas achieving lower MoCA scores than those in urban areas.
- Anaemia is frequent in PD patients, with a prevalence of 58.3% based on haemoglobin values.
- Higher haemoglobin levels are significantly and positively associated with better cognitive performance, suggesting that anaemia is an important, potentially modifiable factor in patients on peritoneal dialysis.

These findings underline the importance of systematic cognitive screening and careful management of anaemia in patients on peritoneal dialysis. Integrating regular cognitive assessment and optimised anaemia treatment into routine clinical practice may help maintain cognitive functioning, improve self-care capacity, and enhance overall quality of life in this vulnerable patient population. Future larger, longitudinal studies are needed to confirm these associations and to develop comprehensive preventive and therapeutic strategies targeting cognitive dysfunction in peritoneal dialysis.

## Figures and Tables

**Table 1:** Sociodemographic characteristics and clinical parameters in patients on peritoneal dialysis.

Variable	n %
Gender	
Male	22 (61.1)
Female	14 (38.9)
Age	
30–44	8 (22.22)
45–59	11 (30.56)
≥60	17 (47.22)
Place of residence	
Rural area	15 (41.7)
Urban area	21 (58.3)
Level of education	
Basic and below	2 (5.6)
Middle	29 (80.5)
Higher and above	5 (13.9)
Marital status	
Married	23 (63.9)
Divorced	13 (36.1)
Comorbidities	
Diabetes mellitus	6 (16.67)
Arterial hypertension	27 (75)
Polycystic kidney disease	3 (8.33)

**Table 2:** Distribution of MoCA scores and prevalence of cognitive dysfunction in patients on peritoneal dialysis.

MoCA	≤25		MoCA	>25	
	n	%		n	%
17	1	2.8	26	4	11.1
20	3	8.3	27	1	2.8
21	3	8.3	28	2	5.6
22	7	19.4	30	4	11.1
23	6	16.7			
24	1	2.8			
25	4	11.1			
Total	25	69.4		11	30.6

**Table 3:** Association between sociodemographic characteristics and MoCA score in patients on peritoneal dialysis.

MoCA	Gender (U) (Male/ Female)	Age (ρ)	Place of Residence (U) (Rural/ Urban)	Education Level (H) (Lower/Higher)	Marital Status (U) (Married/ Other)
**MoCA median** **(Q1–Q3)**	23 (21–26)	-	25 (22–29)	23 (22–26)	23 (22–25)
	23 (22–25)		23 (22–25)	24 (23–26)	24 (22–26)
**Test value**	137.5	−0.48	223.0	5.08	127.5
***p***	0.601	**0.003**	**0.035**	0.166	0.475

Values are presented as median (Q1–Q3). U—Mann–Whitney U test; H—Kruskal–Wallis test; ρ—Spearman correlation. Age was analysed as a continuous variable; therefore, group-based descriptive statistics are not applicable.

**Table 4:** Prevalence of anaemia and erythropoietin therapy in patients on peritoneal dialysis.

Status	n	%
**Haemoglobin**		
Anaemia	21	58.3
No anaemia	15	41.7
**Erythropoietin**		
Not receiving erythropoietin therapy	12	33.3
Receiving erythropoietin therapy	24	66.7

**Table 5:** Pearson and point-biserial correlations between MoCA score, haemoglobin level, and erythropoietin therapy.

Variable 1	Variable 2	Correlation Coefficient	*p*-Value	Type of Correlation
MoCA score	Haemoglobin level	r = 0.487	0.020	Pearson
MoCA score	Erythropoietin therapy	r_pβ_ = −0.124	0.472	Point- biserial
Haemoglobin level	Erythropoietin therapy	r_pβ_ = 0.332	0.048	Point- biserial

Erythropoietin therapy was coded as a dichotomous variable. Pearson correlation was used for associations between continuous variables, while point-biserial correlation (r_pβ_) was used for analyses involving erythropoietin therapy.

## Data Availability

All data relevant to the study are included in the article.

## Abbreviations

The following abbreviations are used in this manuscript:

HD	Hemodialysis
PD	Peritoneal dialysis
CD	cognitive disorders
CKD	Chronic kidney disease
